# Facile Synthesis of FePS_3_ Nanosheets@MXene Composite as a High-Performance Anode Material for Sodium Storage

**DOI:** 10.1007/s40820-020-0381-y

**Published:** 2020-02-18

**Authors:** Yonghao Ding, Yu Chen, Na Xu, Xintong Lian, Linlin Li, Yuxiang Hu, Shengjie Peng

**Affiliations:** 1 Jiangsu Key Laboratory of Materials and Technology for Energy Conversion, College of Materials Science and Technology, Nanjing University of Aeronautics and Astronautics, Nanjing, 210016 People’s Republic of China; 2 Nanomaterials Centre, Australian Institute for Bioengineering and Nanotechnology, and School of Chemical Engineering, The University of Queensland, Brisbane, QLD 4072 Australia

**Keywords:** Anode, Composite, FePS_3_ nanosheets, MXene, Sodium-ion battery

## Abstract

Searching for advanced anode materials with excellent electrochemical properties in sodium-ion battery is essential and imperative for next-generation energy storage system to solve the energy shortage problem. In this work, two-dimensional (2D) ultrathin FePS_3_ nanosheets, a typical ternary metal phosphosulfide, are first prepared by ultrasonic exfoliation. The novel 2D/2D heterojunction of FePS_3_ nanosheets@MXene composite is then successfully synthesized by in situ mixing ultrathin MXene nanosheets with FePS_3_ nanosheets. The resultant FePS_3_ nanosheets@MXene hybrids can increase the electronic conductivity and specific surface area, assuring excellent surface and interfacial charge transfer abilities. Furthermore, the unique heterojunction endows FePS_3_ nanosheets@MXene composite to promote the diffusion of Na^+^ and alleviate the drastic change in volume in the cyclic process, enhancing the sodium storage capability. Consequently, the few-layered FePS_3_ nanosheets uniformly coated by ultrathin MXene provide an exceptional reversible capacity of 676.1 mAh g^−1^ at the current of 100 mA g^−1^ after 90 cycles, which is equivalent to around 90.6% of the second-cycle capacity (746.4 mAh g^−1^). This work provides an original protocol for constructing 2D/2D material and demonstrates the FePS_3_@MXene composite as a potential anode material with excellent property for sodium-ion batteries.

**Electronic supplementary material:**

The online version of this article (10.1007/s40820-020-0381-y) contains supplementary material, which is available to authorized users.

## Highlights

- Few-layered FePS_3_ nanosheets and ultrathin MXene are obtained by liquid ultrasonic exfoliation.
- The novel 2D/2D heterojunction of FePS_3_ nanosheets@MXene composite is synthesized by in situ mixing MXene ultrathin nanosheets with FePS_3_ nanosheets.
- Such unique nanostructure can promote rapid reaction kinetics, prevents electrode pulverization and agglomeration for the volume expansion, and provides the pseudocapacitive contribution.

**Electronic supplementary material:**

The online version of this article (10.1007/s40820-020-0381-y) contains supplementary material, which is available to authorized users.

## Graphical abstract

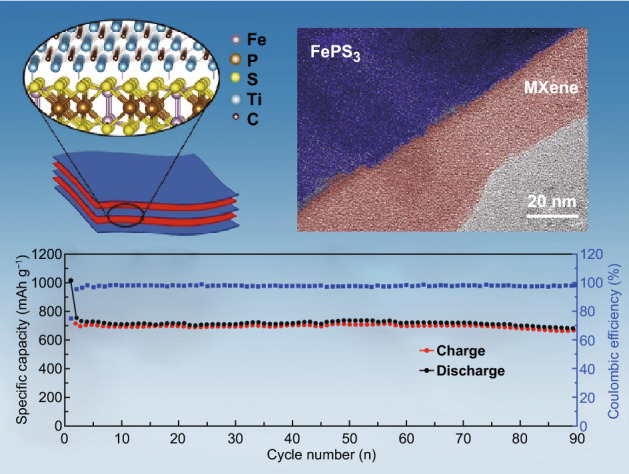

## Introduction

As is known, developing clean and renewable energy sources, such as solar energy, wind energy, and geothermal energy, can reduce the dependence on fossil energy reserves. However, they are easily restricted in practical application by natural conditions [1]. Energy storage system is an effective mean to deal with the practical application problems of intermittent nature energy and improve the utilization efficiency of various energy sources [2]. In the meantime, the development at top speed of consumer electronic devices and electric vehicles has placed higher demands on the energy density and cycle performance [3]. Therefore, developing higher energy density, lower cost, longer cycle performance, and higher efficiency energy storage system is imperative for next generation to overcome the energy shortage problem [4]. Although lithium-ion batteries (LIBs) can meet all those requirements, lithium resources on earth limit the expansion of large-scale energy storage and electric vehicle technology in the future [5]. The exploration and research of sodium-ion batteries (SIBs) as a substitute of LIBs are extremely significant in light of the abundant sodium resources [6]. However, it is the larger ionic radius and higher molar mass of sodium that makes the Na^+^ insertion/extraction kinetics process dilatory and restricts its growth and development. Therefore, searching for appropriate anode materials with high specific capacity, long circulation, and exceptional rate property is very crucial for practical application in the field of stationary energy storage [7, 8].

In 2000, Poizot put forward the mechanism of Li reactivity for transition metal oxides, which is along with the reduction and oxidation process of transition metal, distinguishing from the classical intercalation reaction or the alloying reaction [9, 10]. Owing to much higher theoretical capacity and wider availability than intercalation-based electrodes [11–16] as a result of the multi-electron reaction system [17], conversion reaction-based materials, especially the transition metal sulfides and phosphides, are expected as the anode materials of SIBs [18–23]. Recently, a ternary metal phosphosulfide (MPS_n_) material with particular two-dimensional (2D) layered nanostructure, stacked by interlayer weak van der Waals, was applied as an excellent electrode material for supercapacitors, photocatalyst, electrocatalyst for hydrogen evolution reaction (HER), oxygen evolution reaction (OER), and water splitting, energy storage, and so on [24–30]. Based on the mechanism of conversion reaction, the theoretical capacity of FePS_3_ as an anode material for SIBs exceeds 1300 mAh g^−1^. However, severe volume expansion and dissolution of polysulfides co-occur in the conversion reaction during cyclic process, which cause poor cycling performance.

2D structure is expected as a fully exposed framework for the Na^+^ transport and storage owing to the high specific surface area, which supplies wide and fast access for the insertion and extraction process of Na^+^ with outstanding structural stability. As a result of the excellent structural features and various physicochemical properties, novel 2D materials have attracted the increasing attention in recent years [31], such as graphene [32], graphitic carbon nitride [33], hexagonal boron nitride [34], transition metal dichalcogenides [35], black phosphorus [36, 37], and MXene [38]. Graphene-like 2D nanomaterials MXenes with large redox-active surface area have been extensively used in the energy storage field due to the excellent conductivity [39–41]. There are several advantages presented over others: (1) the exceptional crystalline properties provide lower energy barrier for speedy electron transportation within the atomic layer; (2) the layered structure generates abundant ion diffusion pathways, making the kinetics process more quickly; and (3) excellent strength and toughness can concurrently alleviate the volume stress in the reaction and hold a strong link between each layer [42–45].

In this work, FePS_3_ was employed as the anode material in SIBs to investigate its potential application. The liquid-phase exfoliation of FePS_3_ bulk crystal obtained by solid-state reaction (SSR) method followed by combination with MXene was designed to construct FePS_3_ nanosheets@MXene composite, which can buffer the volume expansion and promote a fast electron/ion transfer. When supplied as the anode of SIBs, a splendid reversible capacity of 676.1 mAh g^−1^ was maintained after 90 cycles at 0.1 A g^−1^ for the FePS_3_ nanosheets@MXene composite. While further improving the current density to 0.5 A g^−1^, a charge capacity of 527.7 mAh g^−1^ was still retained after prolonging to 90 cycles, benefiting from the favorable capacitance kinetics in the charge–discharge process at high-rate process. The resultant 2D/2D hybrid of FePS_3_ nanosheets@MXene is supposed to enhance the electronic conductivity, promote the spread of Na^+^, and buffer the severe volume expansion during cycling. Compared to binary metal sulfide and phosphide for SIBs, the FePS_3_@MXene nanocomposite material demonstrates a competitive and exceptional electrochemical performance, due to the characteristics of novel 2D/2D heterojunction and the phase transformation mechanism. Therefore, the synthetic nanocomposite of FePS_3_@MXene is considered as a promising alternative anode material with superior performance for SIBs.

## Experimental Section

### Chemical Preparation

The following chemicals were used: iron powder (Fe, ≥ 99.9%, Nanjing Crystal Chemical), red phosphorus powder (P, ≥ 98.5%, Energy Chemical, Shanghai), and sublimed sulfur (S, ≥ 99.98%, Nanjing Chemical Reagent). All the reagents and chemicals were used in this research without further purification.

#### Synthesis of Bulk FePS_3_ and Exfoliation of Layered FePS_3_

Typically, bulk FePS_3_ crystals were obtained by heating the mixture of elements in a required stoichiometric ratio (Fe/P/S = 1:1:3) in a sealed quartz ampoule under vacuum (~ 10^−6^ mbar) at 750 °C for 6 days [46]. After a low-temperature solid-state reaction (SSR) process, the products were heated to 500 °C for 2 h in a flowing argon (Ar) atmosphere to remove excess sulfur and red phosphorus. The few layers of FePS_3_ were prepared by exfoliation of bulk crystals. Briefly, 100 mg bulk crystals were dispersed in 100 mL DI water (deionized water, obtained from Milli-Q water purification system) and ultra-sonicated for 8 h in an ice bath. Finally, FePS_3_ nanosheets were collected after freeze-drying.

#### Synthesis of Ti_3_C_2_T_x_ Ultrathin Nanosheets

Multi-layered MXene was obtained by etching of Al from Ti_3_C_2_T_x_. The solution for etching was synthesized through adding 2 g LiF (lithium fluoride) to 30 mL 9.0 M HCl solution (hydrochloric acid) followed by stirring for 5 min. Afterward, 2 g Ti_3_C_2_T_x_ powder was added slowly into the above solution at 40 °C and stirred for 36 h. Then, the suspension was washed with DI water until the value of pH reached greater than 6, sonicated under Ar flow for 2 h, and centrifuged at 3500 rpm for 1 h. Finally, the supernatant was collected and cryopreserved at 4 °C.

#### Synthesis of FePS_3_@MXene Composites

In a typical procedure, 100 mg FePS_3_ nanosheets were dispersed in 100 mL DI water followed by ultrasonication for 30 min to form a uniform solution; then, 3 mL or 6 mL MXene aqueous solution with a concentration of 3 mg mL^−1^ was poured into the solution and stirred for 24 h. After freeze-drying, the products were collected and designated as FePS_3_@MXene-1 and FePS_3_@MXene-2, respectively.

#### Synthesis of NVP/C Powders

Briefly, NVP/C ((Na_3_V_2_(PO_4_)_3_/C, sodium vanadium phosphate/carbon) powder was synthesized by a carbothermal reduction approach. NH_4_VO_3_ and NaH_2_PO_4_·2H_2_O were applied as the reactants, and glucose was employed to provide the carbon, dispersed and mixed in ethanol, and then milled for 24 h in a planetary mill. After fully drying in an oven at 80 °C, the precursor was placed into a corundum boat and calcined under an Ar flow at 900 °C for 4 h.

### Material Characterization

X-ray diffraction (XRD, Bruker D8 Advance, CuKa radiation) was performed to investigate the phase composition. Scanning electron microscopy (SEM, Regulus 8100, 15 kV) and HRTEM (high-resolution transmission electron microscopy, FEI Tecnai G2 F20, 15 kV) were supplied to observe the micro-appearance and the distribution of element with an energy-dispersive X-ray spectroscopy (EDS). X-ray photoelectron spectroscopy (XPS, Escalab 250Xi) was used to explore the elemental composition on the surface and valence states. On the base of BET multipoint approach and BJH model, the pore distribution was characterized by N_2_ adsorption/desorption at 77 K (V-Sorb 2800P). Atomic force microscopy (AFM, Veeco Multimode V) was performed to measure material thickness at room temperature.

### Electrochemical Measurements

All electrochemical tests were measured in a CR2032 model packaged in a glove box (H_2_O, O_2_ < 0.01 ppm) by taking sodium metal as the counter, 1.0 M NaPF_6_ dissolving in ethylene carbonate/diethyl carbonate (EC/DEC) = 1:1 in a volume ratio with 5% fluoroethylene carbonate (FEC) as the electrolyte, and GF/C (glass fibrous membrane, Whatman) as the separator. The anode was produced by a simple slurry process; the as-prepared samples, SWCNTs–COOH (single-walled carbon nanotubes), and CMC (carboxymethylcellulose sodium) were mixed with DI water in a mass ratio of 7:2:1, and then, the mixture was applied onto Cu foil with a coating scraper (MTI, Shenzhen) and then dried at 80 °C overnight. The GCD (galvanostatic charge–discharge) curves were received using a NEWARE measurement system (BTS3000n, Shenzhen) in the potential range of 0.01–3 V (vs. Na^+^/Na) at usual temperature. The cyclic voltammetry (CV) measurements were implemented on an electrochemical workstation (CS2350H). The GITT was tested by discharging or charging the cells for 10 min at 100 mA g^−1^ followed by a relaxation of 60 min. The electrochemical impedance spectroscopy (EIS) tests were performed from the frequency of 100 kHz to 0.01 Hz at 10 mV ac oscillation amplitude under open-circuit voltage status. The mixture of NVP/C, Super-P, and PVDF (polyvinylidene fluoride) in a mass ratio of 7:2:1 with NMP (*N*-methyl-2-pyrrolidone) was coated on an aluminum foil to prepare the cathode for Na^+^ full cell. And the mass ratio of cathode/anode is around 10:1.

## Results and Discussion

**Fig. 1:**
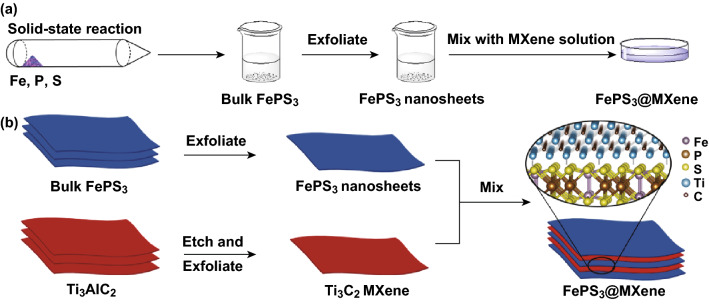
**a** Synthesis process diagram of FePS_3_@MXene. **b** Schematic illustration of MXene assembled on FePS_3_ nanosheets surface and the micromolecular structure in enlarged view

As schematically exhibited in Fig. 1, bulk FePS_3_ crystals were obtained by SSR process. Multi-layered Ti_3_C_2_ MXene was synthesized by selective etching of Al atom from the MAX phase, Ti_3_AlC_2_ (Fig. S1a). In virtue of the unique 2D layered structure property, few-layered FePS_3_ nanosheets and ultrathin MXene can be obtained by liquid ultrasonic exfoliation (Figs. S2 and S1b). Few-layered FePS_3_ nanosheets of thickness 2–3 nm were uniformly coated by ultrathin Ti_3_C_2_ MXene after mixing the FePS_3_ and MXene aqueous solutions, which is denoted as FePS_3_@MXene. By the wet etching approach with in situ HF forming [44–47], MXene can be endowed with high hydrophilicity for the –OH, –O, and –F surface functional groups, which was beneficial to combine with FePS_3_ nanosheets.

**Fig. 2:**
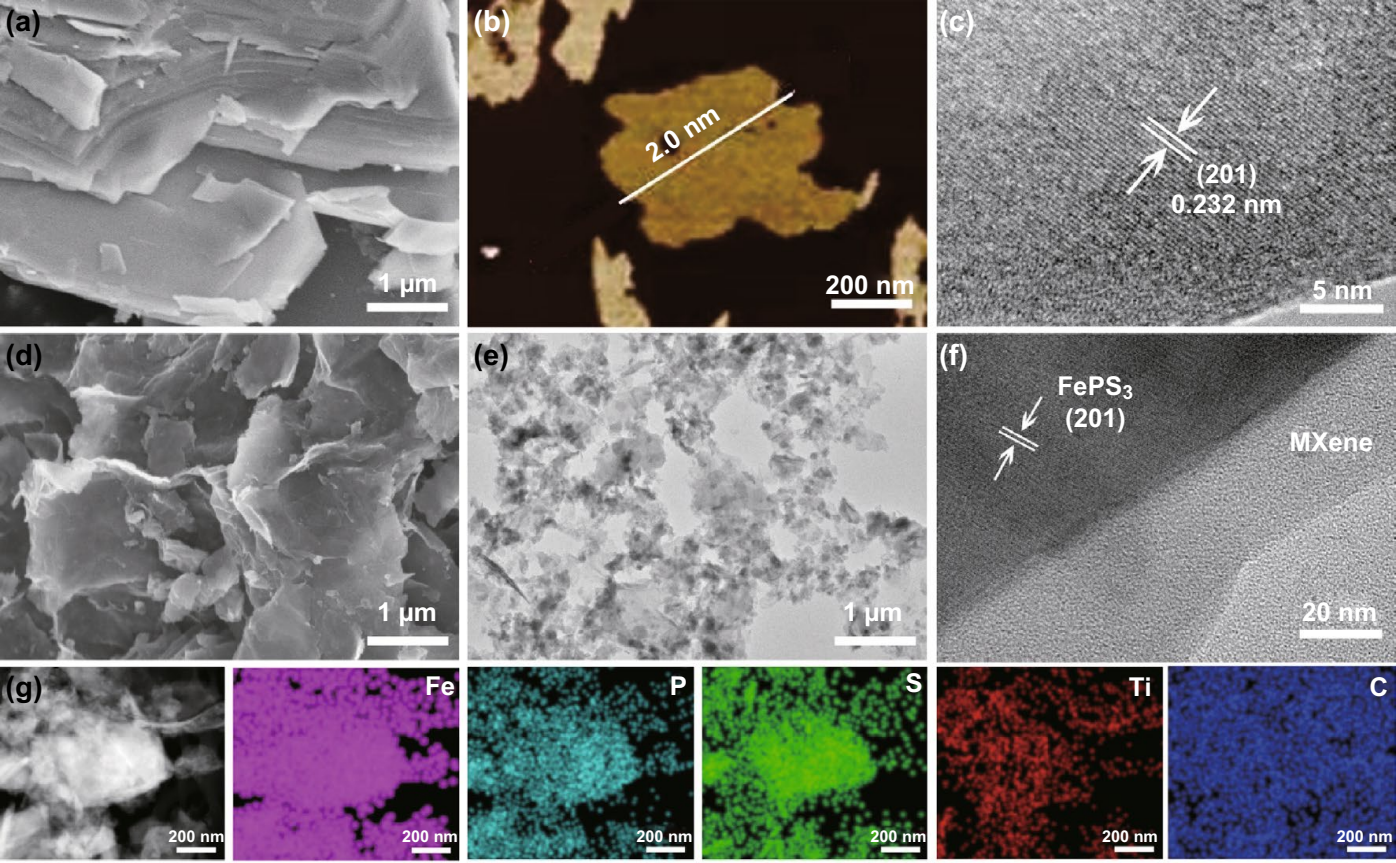
**a** SEM image of bulk FePS_3_. **b**, **c** AFM and HRTEM images of FePS_3_ nanosheets. **d–f** SEM, TEM, and HRTEM images of FePS_3_@MXene. **g** Elemental mapping images of Fe, P, S, Ti, and C

Figure 2a shows the SEM image of bulk FePS_3_ crystal synthesized by SSR step, which is made up of thousands of layers stacked by the weak van der Waals. The corresponding line scan profile analysis of AFM image displayed that the thickness of FePS_3_ nanosheets by liquid exfoliation is found to be around 2.0 nm, indicating 3–4 individual layers in Fig. 2b (the thickness of monolayer is 6.42 Å). Such ultrathin layers lead to high BET surface area of around 14.3 m^2^ g^−1^ (Fig. S3). In Fig. 2c, the clear interlattice indicates the good crystallinity of few-layered FePS_3_ nanosheets, and the distance of 0.232 nm is ascribed to the lattice plane of (201) [48]. The SEM image of FePS_3_@MXene nanocomposite in Fig. 2d reveals the uniform coating of few-layered FePS_3_ nanosheets by ultrathin MXene without obvious bulk crystal in those images. There is no evident difference between FePS_3_ and MXene due to the similar 2D structure and characteristic in Fig. 2e. The EDX analysis of FePS_3_@MXene composite can confirm the presence and homogeneous distribution of Fe, P, S, Ti, C, and F elements, certificating the uniform mixture of the FePS_3_ and Ti_3_C_2_ MXene (Fig. S4). In Fig. 2f, the ultrathin MXene spreads great amorphous phase and the interlattice distance is proved as FePS_3_ phase, which reveals a 2D/2D layered morphology. Through the elemental mapping images exhibited in Fig. 2g, the strong iron, phosphorus, sulfur, titanium, and carbon signals can also illustrate the uniform existence and distribution of FePS_3_ nanosheets and MXene.

**Fig. 3:**
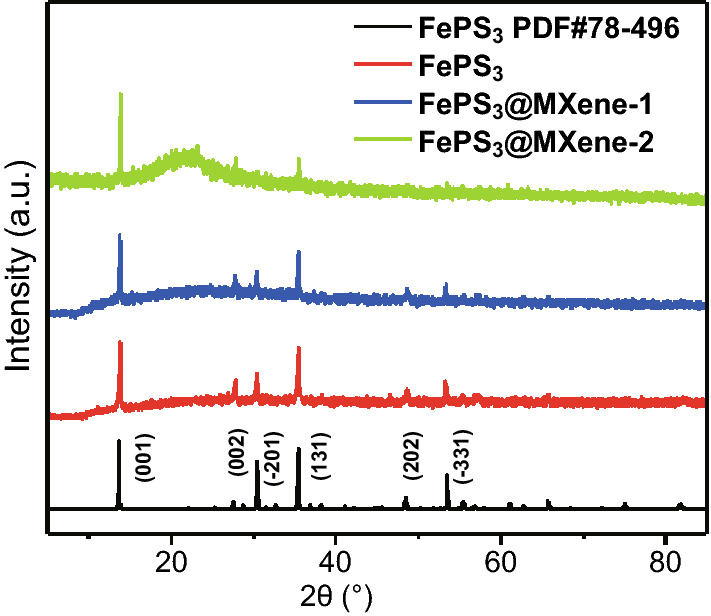
Powder XRD patterns of the synthetic FePS_3_, FePS_3_@MXene-1, and FePS_3_@MXene-2

The phase of samples was initially confirmed by comparing with pure phase XRD patterns as shown in Fig. 3. The XRD patterns of FePS_3_ and FePS_3_@MXene composites show six weak characteristic peaks, which correspond to the (001), (002), (−201), (131), (202), and (−331) planes of FePS_3_ phase (JCPDS No. 78-496). The sharp and intense diffraction peaks of (001) demonstrate the highly crystalline property. It indicates a high purity of samples without impurity peaks. No characteristic diffraction peaks of MXene are observed due to its lower loading content and weak crystallization (Fig. S5).

**Fig. 4:**
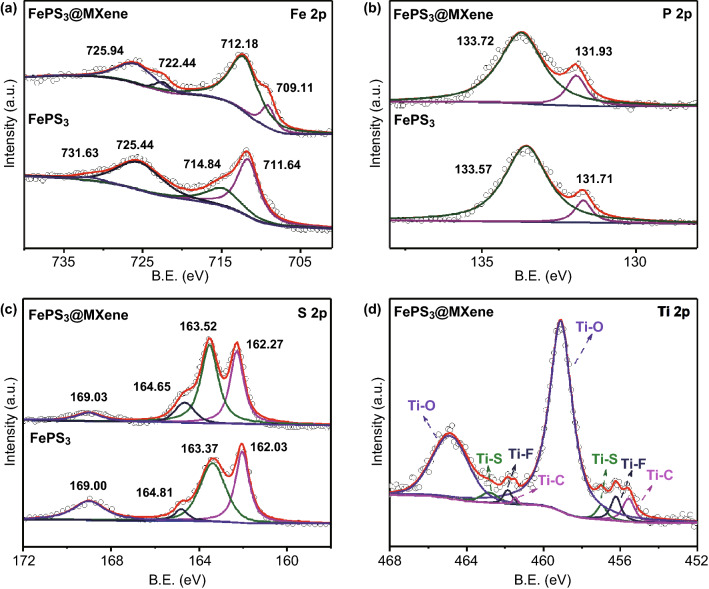
XPS spectra of **a** Fe 2p, **b** P 2p, **c** S 2p, and **d** Ti 2p

XPS analysis further demonstrated the surface elemental composition and valence states. Low-resolution XPS survey verifies the presence of Fe, P, S, O, and C elements in FePS_3_ sample and Fe, P, S, Ti, C, F, and O elements in FePS_3_@MXene-1 sample, respectively (Fig. S6). In Fig. 4a, the deconvoluted high-resolution XPS spectrum of FePS_3_@MXene indicates clearly four peaks in the Fe 2p spectrum. The peaks at 725.94 and 712.18 eV are ascribed to 2p_3/2_ and 2p_1/2_ core levels of Fe^2+^, respectively, while the peaks at 722.44 and 709.11 eV correspond to Fe^3+^. The XPS spectrum of FePS_3_ presents separately two peaks (731.63 and 725.44 eV) at higher binding energies and two peaks (714.84 and 711.64 eV) at lower binding energies in the similar range [24, 25]. The obvious peak shift and area change of Fe 2p spectrum were caused by the connection between the Fe atom and the surface coating of ultrathin MXene. Each spin orbit consists of two satellite peaks with higher binding energy, proving that there is a hybrid between the Fe^2+^, Fe^3+^ levels and the PS_3_ ligand orbit. Furthermore, the XPS spectra of P and S fitted at 2p tracks are similar between FePS_3_ and FePS_3_@MXene. In Fig. 4b, the two splitting peaks located at 131.93 and 133.72 eV were described as the 2p_3/2_ and 2p_1/2_ of the fitted P 2p spectrum, respectively. As exhibited in the deconvoluted S 2p spectrum, the two splitting peaks viewed at 131.71 and 133.57 eV were supposed to 2p_3/2_ and 2p_1/2_ orbitals, respectively. These peaks centered at 164.65 and 169.03 eV were expected to the S 2p and oxidized groups S–O in Fig. 4c. It maintains high consistence between those outcomes with formerly reported data yet. As shown in Fig. 4d, three predominant Ti 2p_3/2_ peaks of FePS_3_@MXene are positioned at 459.10, 456.23, and 455.57 eV, corresponding to the Ti–O, Ti–F and Ti–C bonds. Except for the above three Ti 2p_3/2_ peaks, the peak located at 456.97 eV is attributed to the Ti–S bond between FePS_3_ and MXene, indicating the FePS_3_ nanosheets successfully coated by ultrathin MXene [49].

**Fig. 5:**
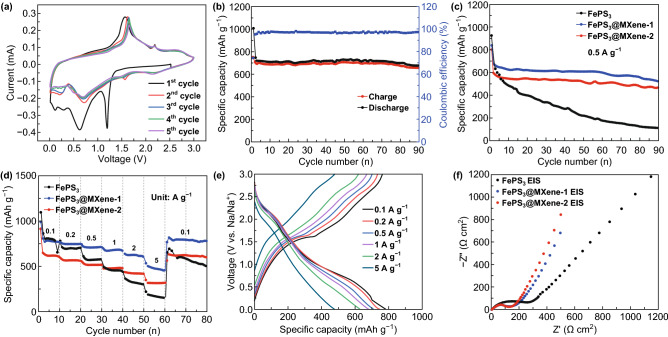
Electrochemical performance comparison for SIBs. **a** CV profiles for the five cycles at 0.1 mV s^−1^. **b**, **c** Cycling stability at 0.1 and 0.5 A g^−1^. **d** Rate capabilities. **e** GCD profiles at various current densities. **f** Nyquist plots

Figure 5a shows the CV profiles of FePS_3_@MXene-1 composite, which was tested at the scan rate of 0.1 mV s^−1^ in the potential range of 0.01–3.0 V. Superior cycling performances of FePS_3_@MXene have been evaluated. As exhibited in Fig. 5b, c, a high reversible capacity (676.1 mAh g^−1^) of FePS_3_@MXene-1 electrode was maintained at 0.1 A g^−1^ after 90 cycles, which corresponds to approximate 90.6% of the second-cycle capacity (746.4 mAh g^−1^), while it still held 527.7 mAh g^−1^ at 0.5 A g^−1^ through 90 cycles. However, pristine FePS_3_ electrode displays poor cycling stability, and a low reversible capacity of 111.3 mAh g^−1^ is retained after 90 cycles at 0.5 A g^−1^. It shows the GCD curves of the 1st, 2nd, 3rd, and 50th, and the first coulombic efficiency is 74.3% (Fig. S7). Figure 5d, e shows the rate performances of FePS_3_, FePS_3_@MXene-1, and FePS_3_@MXene-2. A high reversible capacity of 767, 744, 713, 674, 608, and 449 mAh g^−1^ was delivered by FePS_3_@MXene-1 electrode at the current densities of 0.1, 0.2, 0.5, 1, 2, and 5 A g^−1^, respectively; while the rate returns to 0.1 A g^−1^, the specific capacity of 792 mAh g^−1^ can be retained. Meanwhile, FePS_3_@MXene-2 electrode demonstrates a reversible specific capacity of 610, 568, 517, 476, 418, and 316 mAh g^−1^ at the same current densities. As a contrast, pristine FePS_3_ electrode merely demonstrates the specific capacity of 779, 701, 576, 436, 299, and 154 mAh g^−1^ and pristine ultra-nanosheet MXene barely demonstrates the capacity of 16.3, 10.7, 5.8, 3.6, 2.2, and 1.4 mAh g^−1^ under the same test conditions (Fig. S8), providing minority contribution to the specific capacity.

The FePS_3_@MXene nanocomposite as an anode material for SIBs delivers superior reversible capacity and cyclic stability in this work. The better superior rate performance of the FePS_3_@MXene nanocomposite is attributed to the following factors. (i) MXene restricts the shuttle effect of polysulfides and relieves the dramatic volume expansion in the charge–discharge circulation. (ii) The intercalation of MXene acts as a conductive skeleton, reducing the resistivity of the FePS_3_ nanosheets, which also can be proved by the EIS tests. (iii) Unique 2D skeleton structure provides a large number of active sites and insertion channels, which leads to high-rate performance. In Fig. 5f, the Nyquist plots of the three samples consist of a broad semicircle at the high frequency relating to the charge-transfer-kinetics-controlled section and a straight line at the low frequency representing the mass-transfer-controlled Warburg region, confirming a better electronic and ionic conductivity of FePS_3_@MXene than that of pristine FePS_3_ nanosheets. Adding a small amount of MXene, the slope of the straight line increases obviously and the curvature radius of the semicircle decreases greatly, respectively. Due to the intrinsic characteristic of FePS_3_, the curvature radius of the semicircle has no significant change and the slope of the straight line increases slightly while the proportion of MXene continues to increase. The ultra-small-size active material is uniformly distributed in the conductive MXene throughout the charge and discharge process, thereby ensuring rapid charge transfer for ion and electron.

According to FePS_3_ + 9Na^+^ + 9e^−^ → 3Na_2_S + Fe + Na_3_P, the theoretical capacity of pristine FePS_3_ material is around 1318 mAh g^−1^ for SIBs [50]. It is expected as a potential anode material because of the high theoretical capacity. Based on the intercalation: xNa + FePS_3_ → Na_x_FePS_3_, the sharp peak located at 1.24 V is described to the insertion process of Na^+^ into FePS_3_ monolayer without phase change in the first cathodic scan. This reaction also can be proved by the potential platform in the first discharge curve (Fig. S7), while another wide peak positioned at the range of 0.3–1.0 V, corresponding to the gentle platform in the first discharge curve in Fig. 5a, is assigned to the production of Na_2_S, Na_x_P, and metallic Fe in the conversion reaction, along with the gradual formation of the irreversible solid electrolyte interface (SEI) film. In the subsequent anodic scan, a sharp peak positioned at 2.20 V and a weak peak located at 1.60 V were distinctly displayed, which were related to the oxidation process of the Na_2_S phase and metal Fe, respectively. The following four cathode scans show nearly overlapping CV profiles with two weak peaks at 1.60 and 0.70 V, respectively, corresponding to Na^+^ intercalation into the 2D channel of FePS_3_, the production of Fe and Na_2_S. Similarly, the following three cycles of the GCD profiles are nearly identical, indicating that the electrochemical storage of sodium is stable and reversible [51].

**Fig. 6:**
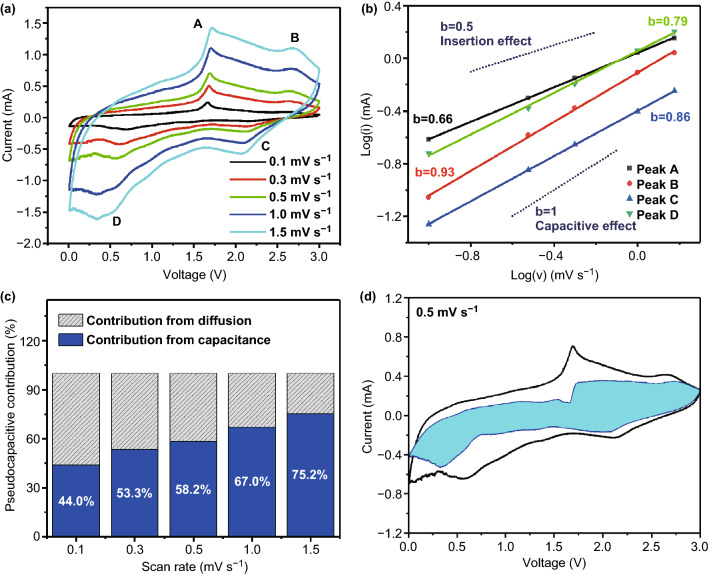
Kinetics analysis for the FePS_3_@MXene-1 electrode. **a** CV profiles at diversity scan speeds. **b** b-value calculation based on the relevance between peak currents and velocity of scanning. **c** Normalized contribution ratio of capacitive and diffusion-controlled capacities. **d** Proportion of capacitive (shaded region) contribution at 0.5 mV s^−1^

To reveal the high cyclic and rate capability of FePS_3_@MXene electrode, the sodium storage mechanism was studied by analyzing CV profiles at diversity scan speeds from 0.1 to 1.5 mV s^−1^ in Fig. 6a. The overall charge storage mechanism of the FePS_3_@MXene electrode can be quantified by dividing the *i* (current) response at a constant *V* (potential) into two mechanisms: *k*_1_*v* (capacitive effects) and *k*_2_*v*^1/2^ (diffusion processes).

$$i(v) \, = a^{v} b = k_{1} v + k_{2} v^{1/2} .$$

Here, *a* and *b* are tunable parameters, *v* means various scan rates, and k_1_ and k_2_ are constants. The value range of *b* is from 0.5 to 1, corresponding to insertion effect and capacitive effect. As depicted in Fig. 6b, the values of *b* for peak A, B, C, and D through calculating the slope of log(*v*)–log(*i*) plots are 0.66, 0.93, 0.86, and 0.79, respectively. Figure 6c exhibits the normalized contribution ratio of capacitive and diffusion-controlled capacities, respectively. When the *v* value is adjusted to 0.5 mV s^−1^, the capacitive contribution ratio reaches 58.2% shown in Fig. 6d, which indicates that the Na^+^ storage mechanism of surface pseudocapacitance contribution should dominate and determine fast and stable sodium storage capability at high current densities.

**Fig. 7:**
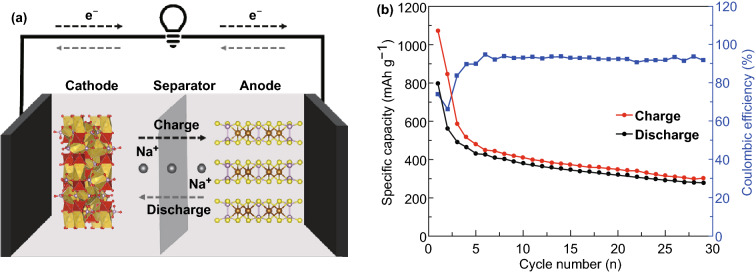
**a** Structure schematic of the full cell. **b** Cycling performance at 0.1 A g^−1^ as to anode

Due to all the advantages of FePS_3_@MXene nanocomposite mentioned above, a full cell of SIBs applying the FePS_3_@MXene nanocomposite as an anode material and NVP/C as a cathode material was assembled and measured to prove the applicability of the FePS_3_@MXene nanocomposite. The NVP/C powder was synthesized by the method reported in the previous literature [52]. The XRD data show that the bulk NVP/C sample exhibits high-purity crystal phase after the comparison (Figs. S9 and S10). The cycle test of full cell was performed at 0.1 A g^−1^ within the potential range of 0.01–3.0 V. The first-cycle charge and discharge capacities of the sodium-ion full cell are 1072 and 796 mAh g^−1^, as shown in Fig. 7b. Due to the capacity balance problem among anode material, cathode material, electrolyte, and system optimization, the capacity is less than the capacity recorded in half-cell. The cycle stability is depicted in Fig. 7b, indicating that the reversible capacity was remained approximately 302 mAh g^−1^ with a coulombic efficiency of 91% after 29 cycles. The energy and power density of the assembled full sodium-ion cell are 424 Wh Kg^−1^ and 131 W Kg^−1^ at 100 mA g^−1^ after 20 cycles, respectively. (The calculation results are based on the quality of the anode material.) According to more sufficient exploration of the entire system, consisting of cathode and electrolyte, the coulombic efficiency and cyclability of the full cell can be further enhanced.

## Conclusions

To summarize, this work examines the potential of ternary FePS_3_ as anode material for SIBs. Through the liquid-phase exfoliation approach and the combination strategy, ultrathin MXene is evenly dispersed onto the few-layered FePS_3_ nanosheets to form FePS_3_@MXene hybrid. The unique 2D/2D heterojunction structure promotes rapid reaction kinetics, prevents electrode pulverization and agglomeration for volume expansion, inhibits the shuttle effect of polysulfides and provides the pseudocapacitive contribution, showing superior rate capacity and cycle stability. Apart from those superiorities of the heterojunction nanostructure, the phase transformation mechanism of pristine FePS_3_ material in essence also imparts the expected electrochemical performance, based on a buffer matrix by the mixed phases for each other. Generally, the work provides a potential anode material FePS_3_@MXene nanocomposite for SIBs through combining the virtues of oriented nanoengineering with the intrinsic phase transformation process of FePS_3_ material.

## Electronic supplementary material

Below is the link to the electronic supplementary material.

Supplementary material 1 (PDF 678 kb)
