# Supplementary material for: Facile Synthesis of FePS3 Nanosheets@MXene Composite as a High-Performance Anode Material for Sodium Storage
Source: Nanomicro Lett. 2020 Feb 18;12:54. doi: 10.1007/s40820-020-0381-y (PMC7770659; doi:10.1007/s40820-020-0381-y)
Supplement: Supplementary file 1 — Supplementary material 1 (PDF 678 kb) [file 40820_2020_381_MOESM1_ESM.pdf]

Supporting Information for

Facile Synthesis of $FePS_3$ Nanosheets@MXene Composite as a High-Performance Anode Material for Sodium Storage

Yonghao Ding[1], Yu Chen[1], Na Xu[1], Xintong Lian[1], Linlin Li[1,*], Yuxiang Hu[2,*], Shengjie Peng[1,*]

[1]Jiangsu Key Laboratory of Materials and Technology for Energy Conversion, College of Materials Science and Technology, Nanjing University of Aeronautics and Astronautics, Nanjing 210016, People's Republic of China

[2]Nanomaterials Centre, School of Chemical Engineering and Australian Institute for Bioengineering and Nanotechnology, The University of Queensland, QLD 4072, Australia

*Corresponding authors. E-mail: lilinlin@nuaa.edu.cn (Linlin Li); y.hu@uq.edu.au (Yuxiang Hu); pengshengjie@nuaa.edu.cn (Shengjie Peng)

Supplementary Figures

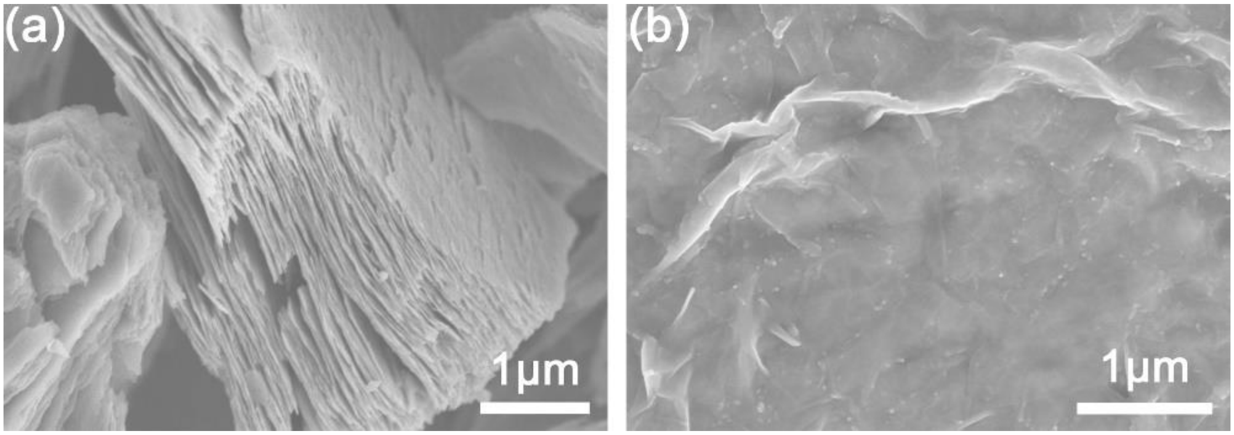

Fig. S1 (**a**) SEM image of multilayered MXene. (**b**) SEM image of few-layered MXene

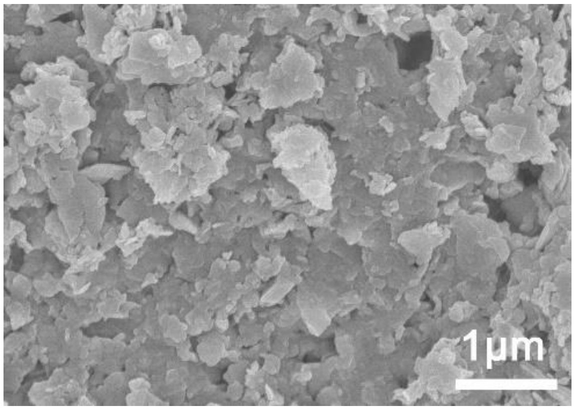

Fig. S2 SEM image of $FePS_3$ nanosheets

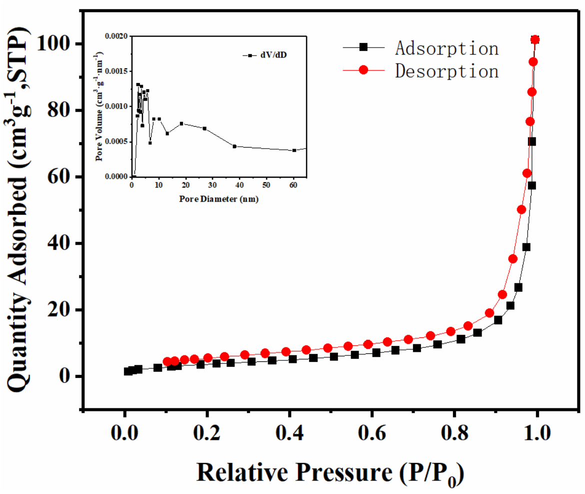

Fig. S3 Typical nitrogen adsorption/desorption isotherms for $FePS_3$ nanosheets. The inset pictures show the pore size distribution calculated by the BJH formula

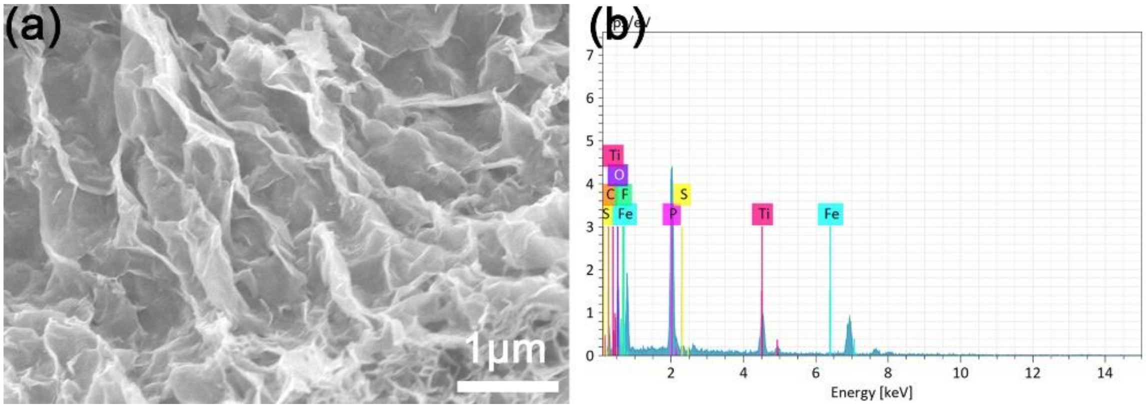

Fig. S4 (**a**) SEM image of $FePS_3$@MXene, (**b**) EDS image of $FePS_3$@MXene

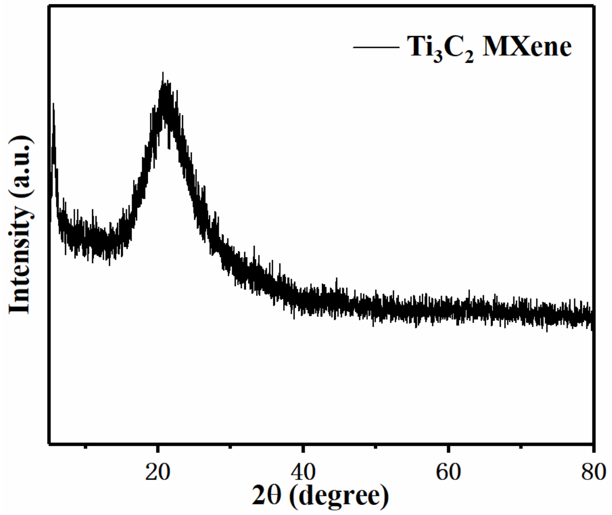

Fig. S5 Powder X-ray diffraction (XRD) patterns of Ti_3C_2 MXene

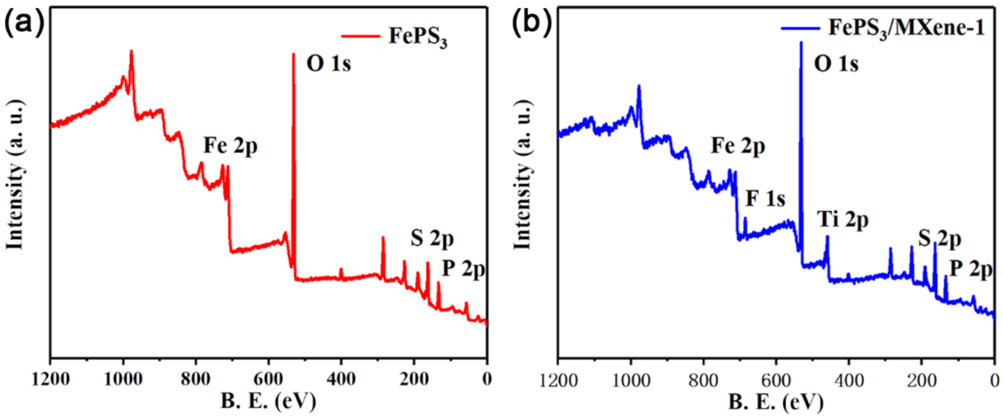

Fig. S6 XPS survey spectrum of the as-prepared $FePS_3$ in the left and $FePS_3$@MXene in the right

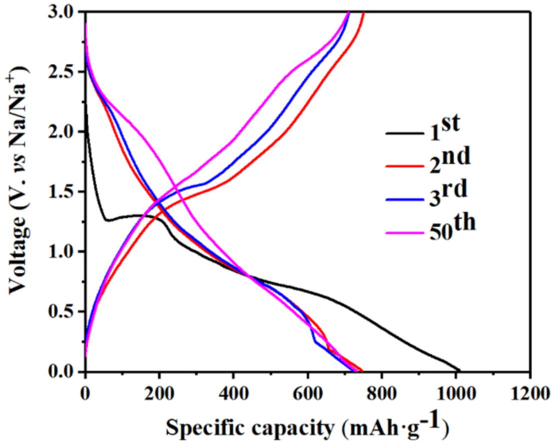

Fig. S7 Charging and discharging curves at 0.1 A g^{-1}

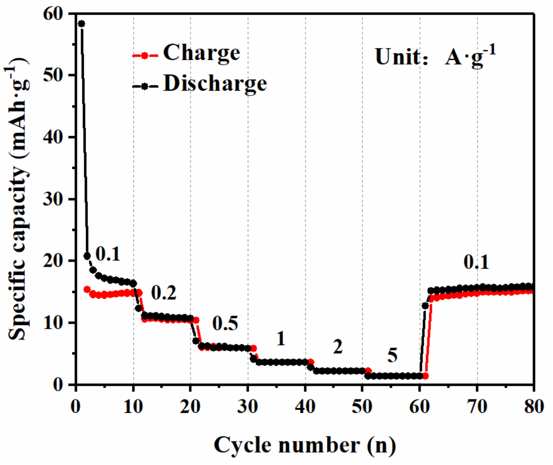

Fig. S8 Rate capabilities of MXene

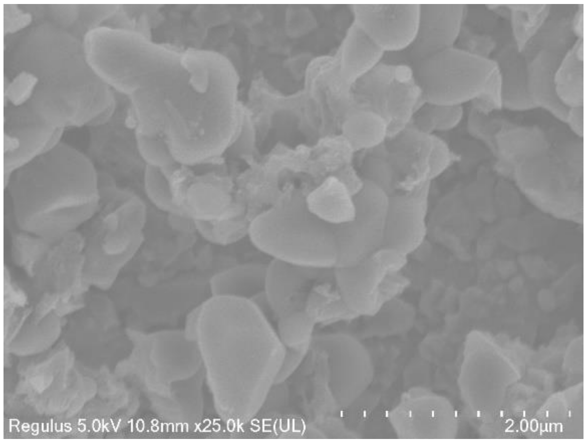

Fig. S9 SEM image of $Na_3V_2(PO_4)_3$

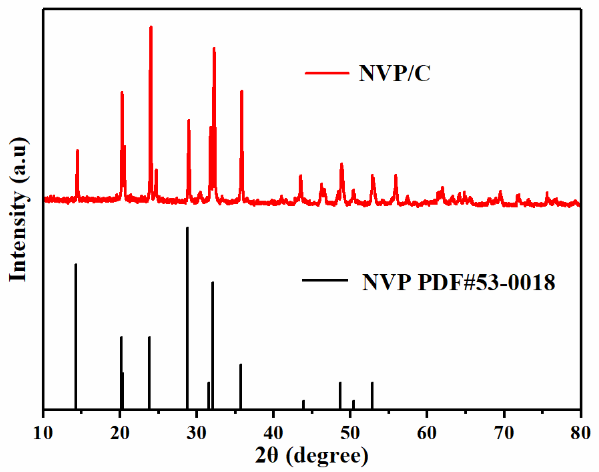

Fig. S10 Powder X-ray diffraction (XRD) patterns of $Na_3V_2(PO_4)_3$

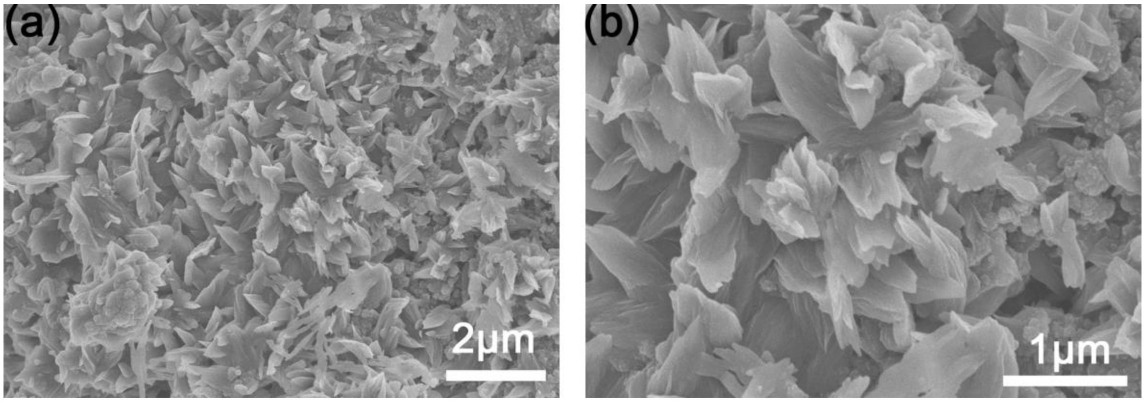

Fig. S11 The SEM images of FePS3@MXene after cycling

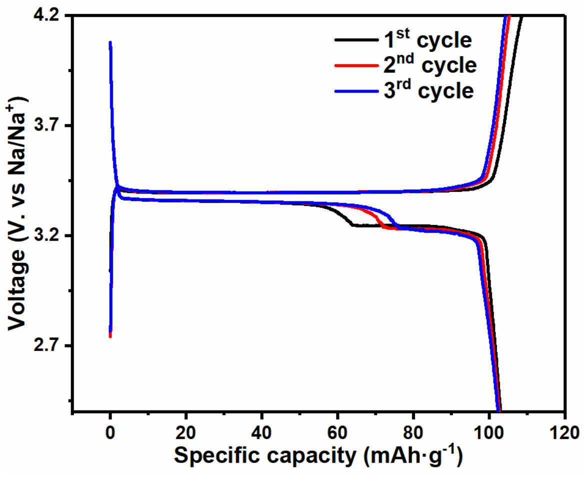

Fig. S12 Charging and discharging curves of $Na_3V_2(PO_4)_3$/C cathode

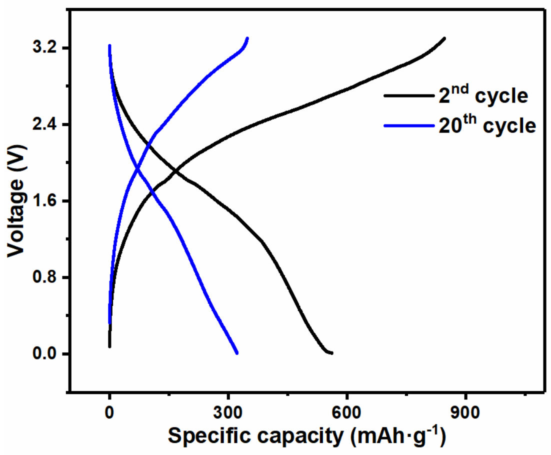

Fig. S13 Charging and discharging curves of the full cell

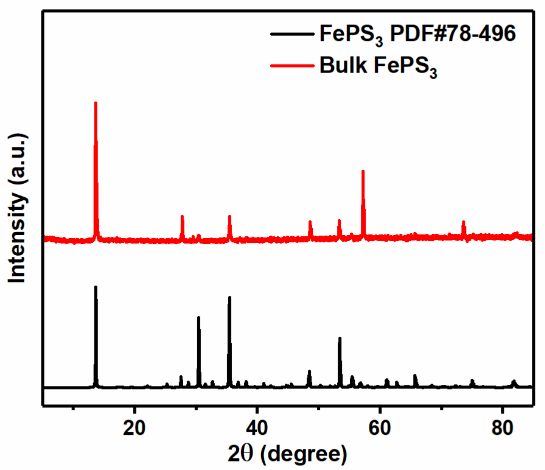

Fig. S14 Powder X-ray diffraction (XRD) patterns of bulk $FePS_3$ crystal
